# Bioinsecticidal activity of *aspergillus*-derived endophytes from *Olea europaea* against *Culex pipiens*: toxicity, histology, and GC-MS profiling

**DOI:** 10.1038/s41598-025-07941-3

**Published:** 2025-07-04

**Authors:** Amr H. Hashem, Wesam M. A. Ward, Amer M. Abdelaziz, Ahmed S. Hashem, Mohamed A. M. Shahat

**Affiliations:** 1 Department of Botany and Microbiology, Faculty of Science, Al-Azhar University, Nasr City, Cairo 11884 Egypt; 2 Zoology and Entomology department, Faculty of Science, Al-Azhar University, Nasr City, Cairo 11884 Egypt; 3 Stored Product Pests Research Department, Plant Protection Research Institute, Agricultural Research Center, Kafr El-Sheikh, Sakha Egypt

**Keywords:** Endophytes, *Aspergillus*, Larvicidal activity, Bioinsecticides, GC-MS analysis, Biological techniques, Environmental sciences, Health care

## Abstract

Endophytic fungi offer a sustainable and eco-friendly alternative to synthetic insecticides, providing targeted pest control with reduced environmental toxicity and lower risk of resistance development. In this study, four endophytic fungi were isolated from *Olea europaea* and their ethyl acetate extracts’ larvicidal efficacy of *Culex pipiens*. Boxplot distributions revealed a positive correlation between metabolite concentration and larval mortality, with higher doses inducing greater lethality. Extracts 1 and 2 exhibited the highest larvicidal activity, with median mortality rates exceeding 60%, while Extracts 3 and 4 demonstrated significantly weaker effects. Dose-response curves further confirmed the potency of Extracts 1 and 2, with lower LC₅₀ values (148.36 and 153.36 µg/mL) compared to Extracts 3 and 4. The histological analysis revealed a dose-dependent impact, with minimal tissue alterations at 25–50 µg/mL, while 150–200 µg/mL caused severe disorganization, apoptosis, and inflammatory responses. The most potent isolates, *Aspergillus niger*(Extract 1) and *Aspergillus flavus*(Extract 2), were identified morphologically and molecularly, with NCBI accession numbers PQ269689 and PQ269690, respectively. GC-MS analysis identified key bioactive compounds, including oleic acid and hexadecanoic acid, supporting their insecticidal potential. These findings highlight *A. niger* and *A. flavus* ethyl acetate extracts as promising sources of bioinsecticides for pest control.

**Supplementary Information:**

The online version contains supplementary material available at 10.1038/s41598-025-07941-3.

## Introduction

Mosquitoes, classified under the order Diptera and family Culicidae, encompass several medically significant genera, including *Aedes*,* Anopheles*, and *Culex*. These hematophagous insects are notorious vectors of deadly pathogens, contributing to the global burden of vector-borne diseases. Notably, malaria, transmitted primarily by *Anopheles* mosquitoes, accounts for over 600,000 deaths annually worldwide, with the majority occurring in sub-Saharan Africa^1^. Given their role in disease transmission, the control of mosquito populations is a cornerstone of public health strategies aimed at mitigating the spread of mosquito-borne illnesses. Conventional mosquito control strategies are multifaceted, encompassing chemical, biological, environmental, and genetic approaches. Chemical control relies heavily on the application of insecticides, including pyrethroids, organophosphates, and carbamates, which target adult mosquitoes and larvae. However, the widespread use of these chemicals has led to the emergence of insecticide resistance, significantly diminishing their efficacy^2^. Biological control methods involve the utilization of natural predators as microbial agents like fungi, which specifically target pathogens without harming non-target organisms^3^. *Olea europaea*, commonly known as the olive tree, belongs to Order: Lamiales, the family Oleaceae. *O. europaea* holds substantial economic, and ecological importance. Ecologically, olive trees contribute to biodiversity in their native habitats and provide habitat for various species^4^. Endophytic fungi are microorganisms that live within the tissues of plants without causing apparent harm. Endophytes are characterized by their ability to produce a variety of bioactive compounds, such as organic acids, phenolic compounds, and antibiotics^5^. In *O. europaea*, several species of endophytic fungi have been identified, these fungi can play significant roles in the health of the olive tree, contributing to its resistance against pathogens and enhancing its growth and stress tolerance^6^. Endophytic fungi hold great potential as a sustainable method for pest control, contributing to integrated pest management and reducing reliance on synthetic chemicals^7^.

The previous study demonstrated that ethyl acetate extracts of *Beauveria bassiana* and *Trichoderma harzianum* possess significant insecticidal activity against *Spodoptera littoralis* and *Aphis gossypii*. Among the two, *B. bassiana* extract exhibited higher toxicity toward both pests, particularly against *S. littoralis*, while *T. harzianum* extract showed stronger efficacy against *A. gossypii*^8^. The symbiotic relationships between endophytic fungi and their plant hosts not only contribute to the ecological resilience of the plants but also offer a vast repository of bioactive metabolites with potential applications in agriculture, medicine, and biotechnology^9^. In the field of pest management, endophytic fungi produce insecticidal components like beauvericin, bassianolide, and destruxins, which disrupt insect physiology by targeting neural pathways, inhibiting enzyme activity, or impairing cellular functions^10^. Their role in mosquito control extends beyond direct toxicity, as endophytic fungi contribute through multiple mechanisms. Numerous fungal species synthesize mycotoxins, alkaloids, and terpenoids that interfere with larval physiology and development. Compounds such as beauvericin, destruxins, and trichothecenes have been identified as potent larvicidal agents, demonstrating the effectiveness of endophytic fungi in disrupting mosquito life cycles and supporting sustainable vector control strategies^11^. Certain fungi produce chitinases and proteases that degrade the protective cuticle of mosquito larvae, leading to dehydration and mortality. Additionally, fungal metabolites can disrupt the gut microbiome of mosquito larvae, impairing digestion and nutrient absorption^12^. Some endophytes generate reactive oxygen species (ROS) that cause cellular damage and apoptosis in mosquito larvae^13^.

A compelling example of this is the *O. europaea*, a species native to the Mediterranean region and economically significant for its oil-rich fruits^14^. The olive tree hosts a diverse community of endophytic fungi that play essential roles in enhancing plant health, stress tolerance, and resistance to pathogens^15^.

Secondary metabolites, including alkaloids, polyketides, and terpenoids, from *Aspergillus* species have shown significant potential as plant growth regulators and insecticidal agents that can influence plant development by modulating hormone-signaling pathways, enhancing stress tolerance, and promoting root growth^16^.

Therefore, the primary aim of this study is to isolate endophytic fungi from *O. europaea* and evaluate their potential as biocontrol agents against *Culex pipiens* larvae. By characterizing the bioactive secondary metabolites produced by these fungi, the study seeks to identify novel larvicidal compounds that can be utilized in sustainable mosquito control strategies. Additionally, this study aims to evaluate the toxicity of these fungi by determining the lethal concentration required to kill 50% of the larvae (LC_50_) and investigating their histological effects on mosquito tissue to explore the mechanisms by which these endophytic fungi exert their larvicidal effect.

## Materials and methods

### Isolation of endophytic fungi

The olive tree, *Olea europaea*, plants were collected from the Botany and Microbiology Department, Faculty of Science, Al-Azhar University, Cairo, Egypt (30 º03’15.48’’N, 31 º19’12.75’’E). Experimental research and field studies on plants, including the collection of plants and identification, comply with relevant institutional, national, and international guidelines and legislation by Prof. Dr. Abdou Marie Hamed at the Botany and Microbiology Department, Faculty of Science, Al-Azhar University, Cairo, Egypt. Healthy leaves of *Olea europaea* were thoroughly washed and sterilized using tap water, 70% ethanol, and 4% sodium hypochlorite. The sterilized leaves were then placed on PDA-chloramphenicol (0.2 g/L). To prepare the PDA medium, 250 g of potatoes were washed, cut into cubes, and boiled in 500 ml of water for 30 min. Then, 15 g of agar was melted in 500 ml of water. The potato mixture was filtered through filter paper into the flask containing the melted agar. After adding 20 g of dextrose and mixing well, the solution was adjusted to 1 L with water. The potato dextrose broth (PDB) had the same gradient without agar. The PDA was sterilized by autoclaving at 121 °C for 15 min. The cultured plates were incubated at 27 °C ± 2 °C for 21 days, with daily observations. Hyphal tips emerging from the cultured leaf segments were subsequently sub-cultured onto PDA^17^.

### Extraction of active metabolites

For 21 days, endophytic fungi were grown in 1 L of PDB at 27 °C ± 2 °C under static conditions. Filtration was used to remove the spores and mycelia from the fermenting broth. EtOAc (1:1) was used twice to extract the culture filtrates of the isolated fungal endophytes. The mixture was shaken until two distinct, clear layers appeared. A separating funnel was used to separate the aqueous layer from the EtOAc layer. Air drying was used to evaporate the collected EtOAc layer at 40 °C. The fungal crude extract was dissolved using DMSO at a concentration of 1 mg/mL and then stored at -20 °C until further experiments were conducted^18^.

### Larvae collection and rearing

Mosquito strain used in this study was maintained over multiple generations in the Medical Entomology Insectary at the Animal House, Faculty of Science, Al-Azhar University. The insects were reared under controlled environmental conditions (27 ± 2 °C, 70 ± 10% RH) and a 14:10-hour light/dark photoperiod. Importantly, the colony was maintained without prior exposure to insecticides to ensure baseline susceptibility. Adult mosquitoes were housed in wooden cages following their emergence. They were provided with sponge pieces soaked in a 10% sucrose solution daily for 3 to 4 days to ensure adequate nutrition. After this period, female mosquitoes were allowed to feed on blood of pigeons, a necessary step to stimulate egg production and ensure colony continuity^19^.

### Larval toxicity bioassay

The larvicidal activities of metabolites isolated from four endophytic fungi (designated as Extract 1 (FOE1), Extract 2 (FOE2), Extract 3 (FOE3), and Extract 4 (FOE4)) were evaluated using a slightly modified protocol based on established methodologies^20^. Bioassays were conducted using five concentrations of the fungal metabolites (25, 50, 150, and 200 µg/mL). For each treatment, 1st to 4th instar larvae were introduced into sterile borosilicate glass beakers containing 100 mL of distilled water and the specified metabolite concentrations. Each concentration was tested in triplicate, with a total of 150 larvae per treatment. During the experiment, larvae were not provided with food to eliminate potential confounding factors. Mortality rates were recorded after 48 h of exposure. Control groups (10% DMSO and deionized water) were included in triplicate for each treatment condition. Larval mortality was calculated in triplicates; mortality (%) was corrected via; Abbott’s formula^21^. Lethal concentrations (LC_50_ and LC_90_) were determined using probit analysis^22^which statistically assessed the dose-response relationship.

### Histology

Histological analysis of the digestive system was conducted on third instar larvae, including both treated and control groups. The larvae were initially fixed in 2.5% glutaraldehyde prepared in 0.1 M sodium cacodylate buffer (pH 7.4) for 4 h. Following fixation, the samples were dehydrated through a graded ethanol series (70%, 80%, 90%, 96%, and 100%), with each concentration applied for 15 min. The dehydrated larvae were then embedded in Historesin JB4 to facilitate sectioning. Using a microtome, the embedded samples were sliced into a series of 3-µm-thick sections. These sections were stained with hematoxylin–eosin to enhance tissue contrast and subsequently examined under a light microscope. Micrographs were captured to document the histological features and any morphological alterations induced by the treatment^23^. All images were acquired using an Olympus stereo microscope SZX10 equipped with an Olympus DP72 camera.

### Identification of endophytic fungi

Morphological characteristics, including colony color, texture, and diameter, were examined for the endophytic fungi and molecular identification techniques were used for further characterization. The primer sequences employed were Reverse ITS2-R (5’-TCCTCCGCTTATTGATATGC-3’) and Forward ITS1-F (5’-TCCGTAGGTGAACCTGCGG-3’). The PCR products were purified using the Gene JET PCR Purification Kit (Thermo K0701), following the manufacturer’s instructions. Sequencing of these products was conducted with an Applied Biosystems sequencing-ready reaction kit from Foster, CA, USA. The resulting sequences were then submitted to the BLAST algorithm at NCBI to find closely related phylogenetic sequences. The evolutionary history was inferred using the Neighbor-Joining method with distances calculated by the Maximum Composite Likelihood method, expressed as base substitutions per site. Branch lengths reflect these distances. Proportions of unambiguous sites are indicated at internal nodes. All evolutionary analyses were performed using MEGA11 software. Finally, the fungal isolates were added to the NCBI database with new accession numbers^24^.

### Identification of active metabolites by GC mass analysis

The identification, enumeration, and analysis of metabolites were conducted using Gas Chromatography–Mass Spectrometry (GC-MS). Initially, the crude extract was prepared by dissolving it in spectroscopic-grade methanol to ensure purity and compatibility with the analytical technique. The GC-MS analysis was carried out using a Thermo Scientific Trace GC1310-ISQ mass spectrometer (Austin, Texas, USA), which was equipped with a direct capillary column measuring 30 m in length, with a film thickness of 0.25 μm and an internal diameter of 25 mm. Helium served as the carrier gas, and a 1 µL aliquot of the sample was injected at an inlet temperature of 250 °C, utilizing a split ratio of 1:30 to achieve optimal sample introduction. The oven temperature was initially maintained at 50 °C for five minutes and then gradually increased to 230 °C. The mass spectrometer operated in electron ionization (EI) mode at 200 °C with an ionization energy of 70 eV, scanning ions in the mass-to-charge (m/z) range of 40 to 1000. Spectral data obtained were matched against reference libraries, including NIST 11 and WILEY 09 (Wiley, New York, NY, USA), to identify the chemical structures, molecular weights, and names of the detected compounds with high confidence^25^.

### Statistical analysis

The larval mortality rate was calculated using Abbott’s formula to correct for control group mortality. The concentration-response assay data were subjected to probit analysis using IBM SPSS Statistics version 20 software to determine the lethal concentrations (LC_50_ and LC_90_). Prior to statistical analysis, all data were evaluated for compliance with the assumptions required for parametric models. The Shapiro-Wilk test was employed to assess the normality of residual data distribution, while the Bartlett test was used to verify the homogeneity of variances. Subsequently, one-way analysis of variance (ANOVA) was conducted to identify significant differences in mortality rates among the experimental embryo/larvae groups. Additionally, Pearson’s correlation coefficients were calculated to evaluate relationships between variables, and linear regression analysis was performed to further explore dose-response relationships.

## Results

### Toxicity assay

**Fig. 1:**
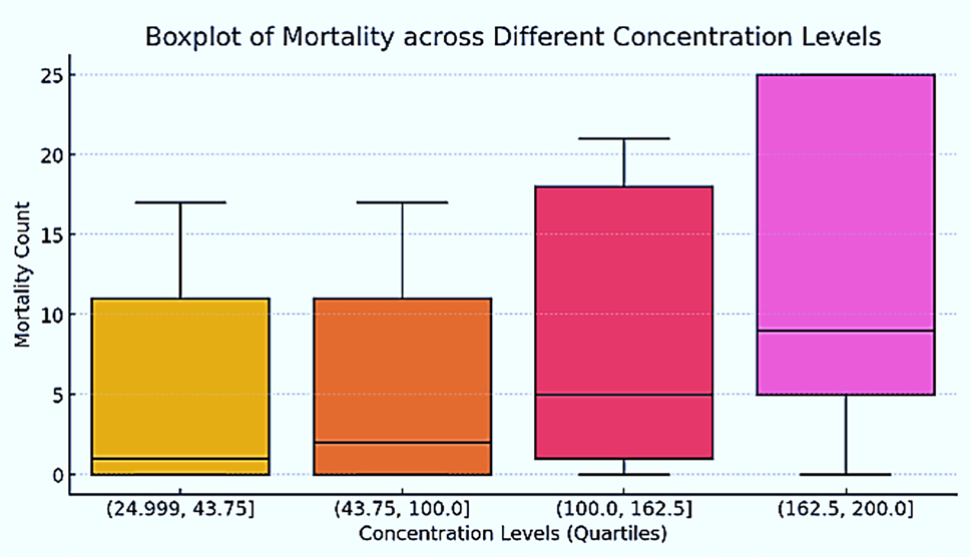
Dose-dependent increase in larval mortality of *Culex pipiens* exposed to ethyl acetate extracts of Aspergillus spp. across concentration quartiles.

Distribution of larval mortality counts across four quartile-based concentration ranges of the tested endophytic metabolites was illustrated as a boxplot (Fig. 1). The data indicate a trend of increasing mortality with higher concentration levels. At the lowest concentration range (24.999, 43.75)(24.999, 43.75)(24.999,43.75), mortality counts exhibit a relatively narrow interquartile range (IQR) suggesting minimal larvicidal effect at these lower doses. The second quartile range (43.75,100.0)(43.75, 100.0)(43.75,100.0) shows a slightly wider spread, but the median mortality remains low, indicating only a marginal increase in lethality compared to the first group. A more pronounced rise in mortality is observed in the third quartile range (100.0, 162.5) (100.0, 162.5)(100.0,162.5), where the median mortality count is notably higher than the preceding groups. The interquartile range is also broader, suggesting increased variability in response. The highest concentration range (162.5, 200.0), (162.5, 200.0), (162.5, 200.0) demonstrates the most significant larvicidal effect, with the median mortality count substantially elevated. The upper whisker extends to the highest recorded mortality values, reinforcing the dose-dependent response observed across the concentration groups. Overall, the boxplot supports a positive correlation between metabolite concentration and larval mortality, with higher concentrations leading to greater mortality and increased variability in response. These findings align with the concentration-response relationship typically observed in toxicological studies.

**Fig. 2:**
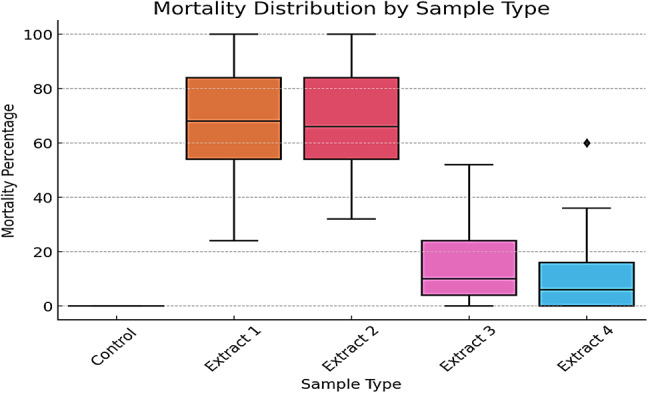
Comparative larvicidal efficacy of Aspergillus-derived extracts against *Culex pipiens larvae*.

On the other hand, distribution of larval mortality percentages across different treatment groups, including a control group and four extracts, was estimated as a boxplot (Fig. 2). The control group exhibited no mortality, confirming the absence of external lethal factors unrelated to the tested extracts. Among the treatment groups, Extract 1 and Extract 2 demonstrated the highest larvicidal activity, with median mortality rates exceeding 60%. Both extracts displayed a broad interquartile range (IQR), indicating variability in response, with minimum values around 30% and maximum values reaching 100%. This suggests a potent yet somewhat variable effect of these extracts on larval mortality. In contrast, Extract 3 and Extract 4 resulted in substantially lower mortality rates. Extract 3 exhibited a median mortality below 20%, with a wider spread of data points and an outlier suggesting occasional higher lethality. Extract 4 demonstrated the weakest larvicidal effect, with a median mortality close to 10% and most values clustering near the lower end of the mortality scale. Overall, the results suggest a significant variation in the larvicidal efficacy of the tested extracts, with Extract 1 and Extract 2 being the most effective, while Extract 3 and Extract 4 exhibited limited toxicity. These findings indicate that the bioactivity of the extracts is concentration- and composition-dependent, warranting further investigation into their chemical constituents and mechanisms of action.

**Fig. 3:**
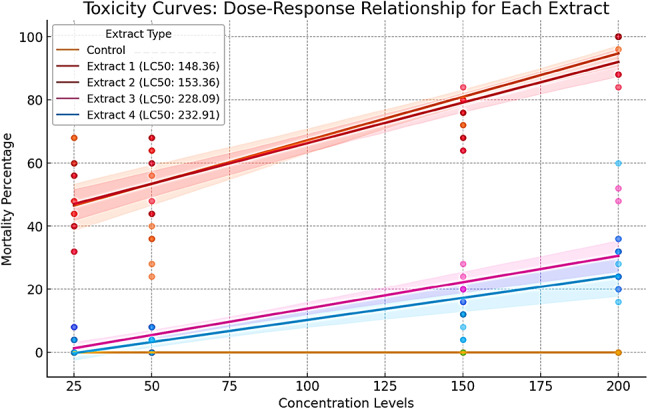
Dose-response curves and LC₅₀ values of fungal extracts against *Culex pipiens* larvae.

The dose-response curves of the relationship between extract concentration and larval mortality percentage were presented (Fig. 3). The data indicate a concentration-dependent increase in mortality across all tested extracts, with varying degrees of toxicity. The control group showed negligible mortality across all concentration levels, confirming that larval death was attributable to the applied extracts rather than external factors. Among the tested extracts, Extract 1 and Extract 2 demonstrated the highest toxic effects, as indicated by their lower LC₅₀ values (148.36 and 153.36 µg/mL, respectively). These extracts exhibited steep dose-response slopes, suggesting a strong larvicidal activity even at lower concentrations. In contrast, Extract 3 and Extract 4 displayed significantly weaker toxic effects, with LC₅₀ values of 228.09 and 232.91 µg/mL, respectively. Their dose-response slopes were more gradual, indicating a less pronounced increase in mortality with rising concentration levels. The 95% confidence intervals, represented by the shaded regions around each curve, highlight the variability in mortality responses. Extract 1 and Extract 2 exhibit tighter confidence bands, suggesting more consistent toxic effects, whereas Extract 3 and Extract 4 display wider confidence intervals, indicating higher variability in their impact. Overall, these findings confirm that the tested extracts exhibit differential larvicidal potency, with Extract 1 and Extract 2 emerging as the most effective candidates. The observed trends underscore the potential of these extracts for further toxicological and bioactivity studies.

### Histology observations

**Fig. 4:**
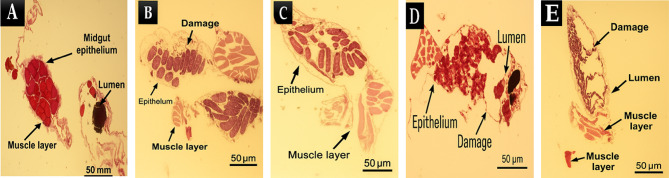
Histological evaluation of muscle fiber integrity in control (A) and treated groups (B. Extract 1, C. Extract 2, D. Extract 3, E. Extract 4).

The histological examination of digestive system tissues treated with fungal extracts at concentrations of 25, 50, 150, and 200 µg/mL revealed a dose-dependent effect on tissue morphology, cellular integrity, and inflammatory response (Fig. 4). At lower concentrations (25 and 50 µg/mL), the tissue architecture remained largely intact, with minimal structural alterations and preserved epithelial integrity. Cellular morphology appeared normal, with well-defined nuclei and cytoplasmic boundaries, suggesting minimal cytotoxicity at these doses. In contrast, at higher concentrations (150 and 200 µg/mL), significant histopathological changes were observed.

**Fig. 5:**
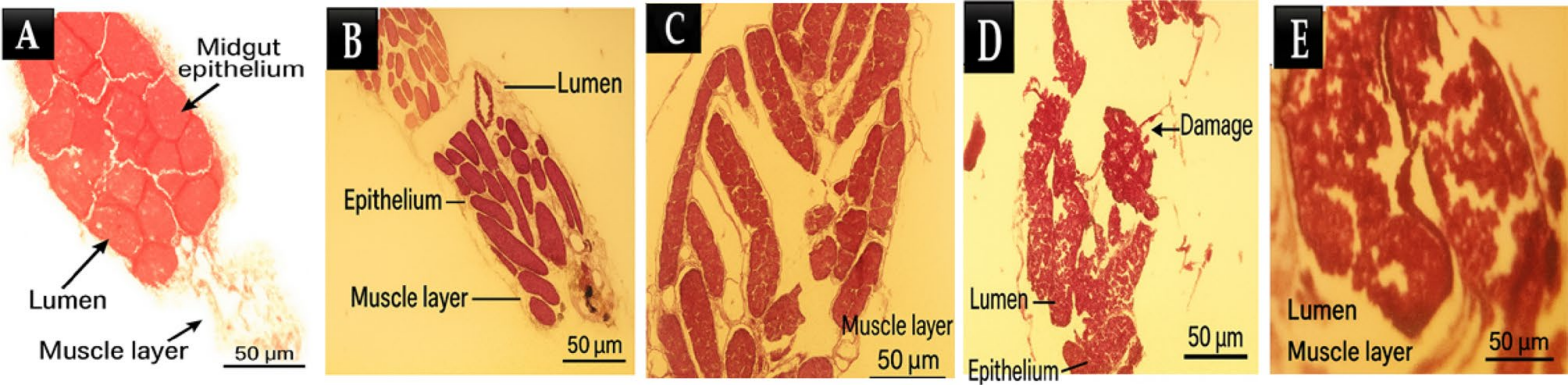
Toxicity-Induced morphological changes in digestive tissue across varying extract doses. Control (A) and Treated Groups (B. Extract 1, C. Extract 2, D. Extract 3, E. Extract 4).

The epithelial layer exhibited noticeable disorganization, with increased cellular swelling, nuclear condensation, and signs of apoptosis (Fig. 5). These alterations were more pronounced at 200 µg/mL, where extensive structural disruption, loss of cellular cohesion, and necrotic regions were evident. Additionally, a dose-dependent increase in immune cell infiltration was observed, indicating a progressive inflammatory response. While minimal inflammatory infiltration was detected at 25 µg/mL, moderate to severe immune cell accumulation was noted at 150 and 200 µg/mL, suggesting a direct relationship between extract concentration and tissue inflammation. Quantitative analysis of the histological sections further supported these observations. The number of detected cellular structures decreased progressively with increasing concentrations of fungal extract, suggesting a reduction in viable cell populations at higher doses. The estimated inflammatory response score indicated mild tissue stress at 25–50 µg/mL, whereas a significant immune reaction was observed at 150–200 µg/mL. Similarly, necrotic changes were negligible at lower concentrations but became increasingly evident at higher doses, with severe tissue degeneration at 200 µg/mL.

### Identification of most potent endophytic fungi

**Fig. 6:**
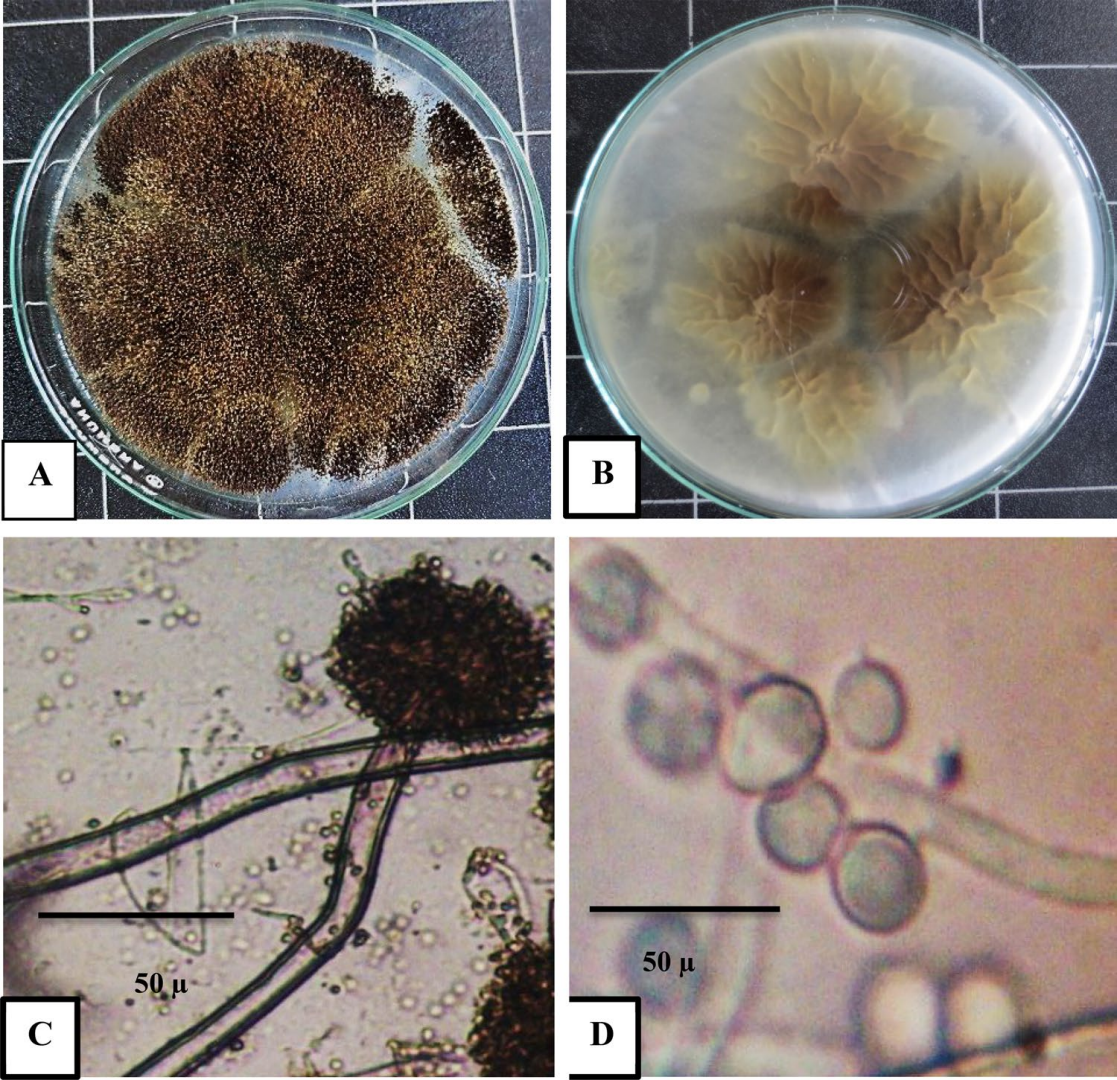
Morphological characters of *Aspergillus niger;* (**A**) colony on PDA, (**B**) reverse colony on PDA, (**C**) conidiophore and conidial head mag 10 × 40, and (**D**) conidia mag 20 × 40.

From the previous experiments, the most potent endophytic fungi were Extract 1, and Extract 2, thus it were identified morphologically and molecular as the following; Extract 1: Morphologically identified as *Aspergillus niger*, colonies grow quickly, reaching 30 mm in diameter in 3 days at 27 °C on PDA. Figure 6A,B illustrate the colonies’ black color and pale yellow reverse color, respectively. Conidiophores had a brown hue and culminated in radiating vesicles that were mainly covered in two series of sterigmata (Fig. 6C). The round, spiny-walled brown conidia with a brownish-black conidial head are shown in Fig. 6D.

**Fig. 7:**
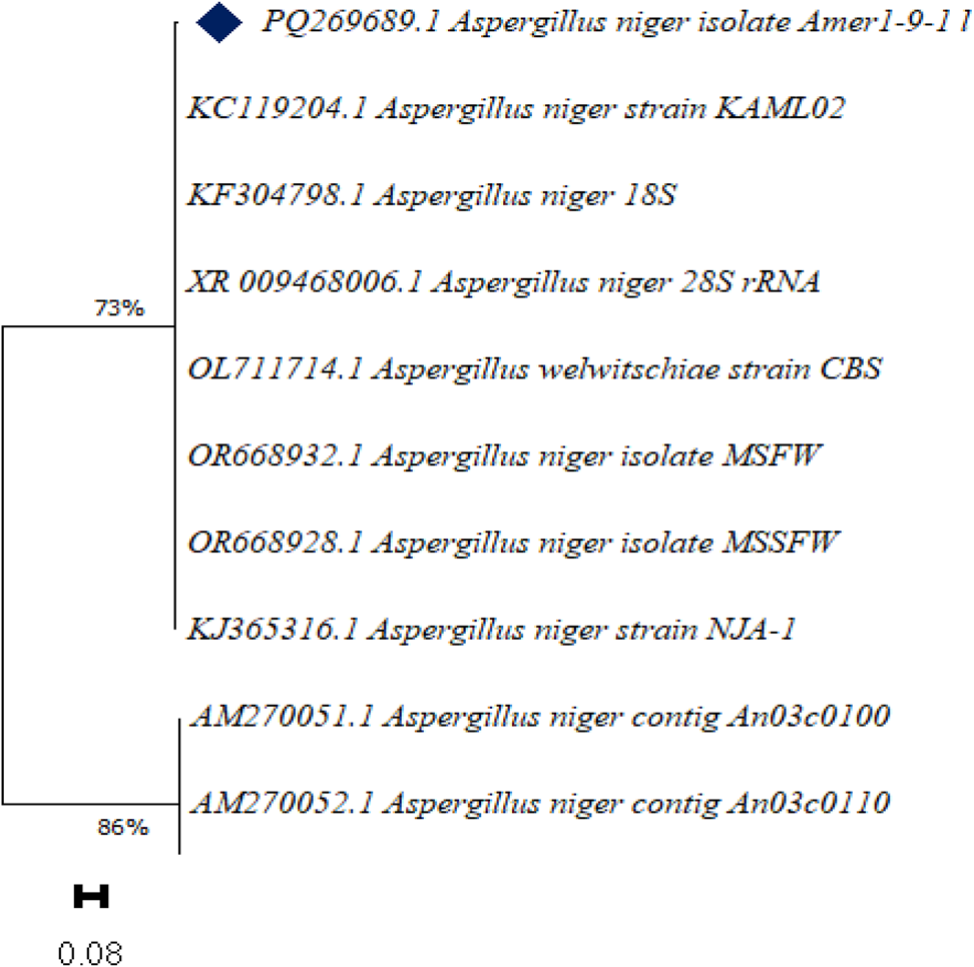
Phylogenetic tree of *Aspergillus niger*. The evolutionary history was inferred using the Neighbor-Joining method with distances calculated by the Maximum Composite Likelihood method, expressed as base substitutions per site. Branch lengths reflect these distances. Proportions of unambiguous sites are indicated at internal nodes. All evolutionary analyses were performed using MEGA11 software.

To confirm the morphological identification, molecular identification was conducted. Subsequently, the strain was deposited in the NCBI with accession number PQ269689.1 Its similarities to *Aspergillus niger* strain KAML02 (KC119204.1), *Aspergillus niger* 18 S (KF304798.1), *Aspergillus niger* 28 S (XR009468006.1), *Aspergillus welwitschiae* strain CBS (OL711714.1), *Aspergillus niger* isolate MSFW (OR668932.1), *Aspergillus niger* isolate MSSFW (OR668928.1), *Aspergillus niger* strain NJA-1 (KJ365316.1), *Aspergillus niger* contig An03c0100 (AM270051.1), *Aspergillus niger* contig An03c0110 (AM270052.1) with 98.94% (Fig. 7 and Table 1S).

**Fig. 8:**
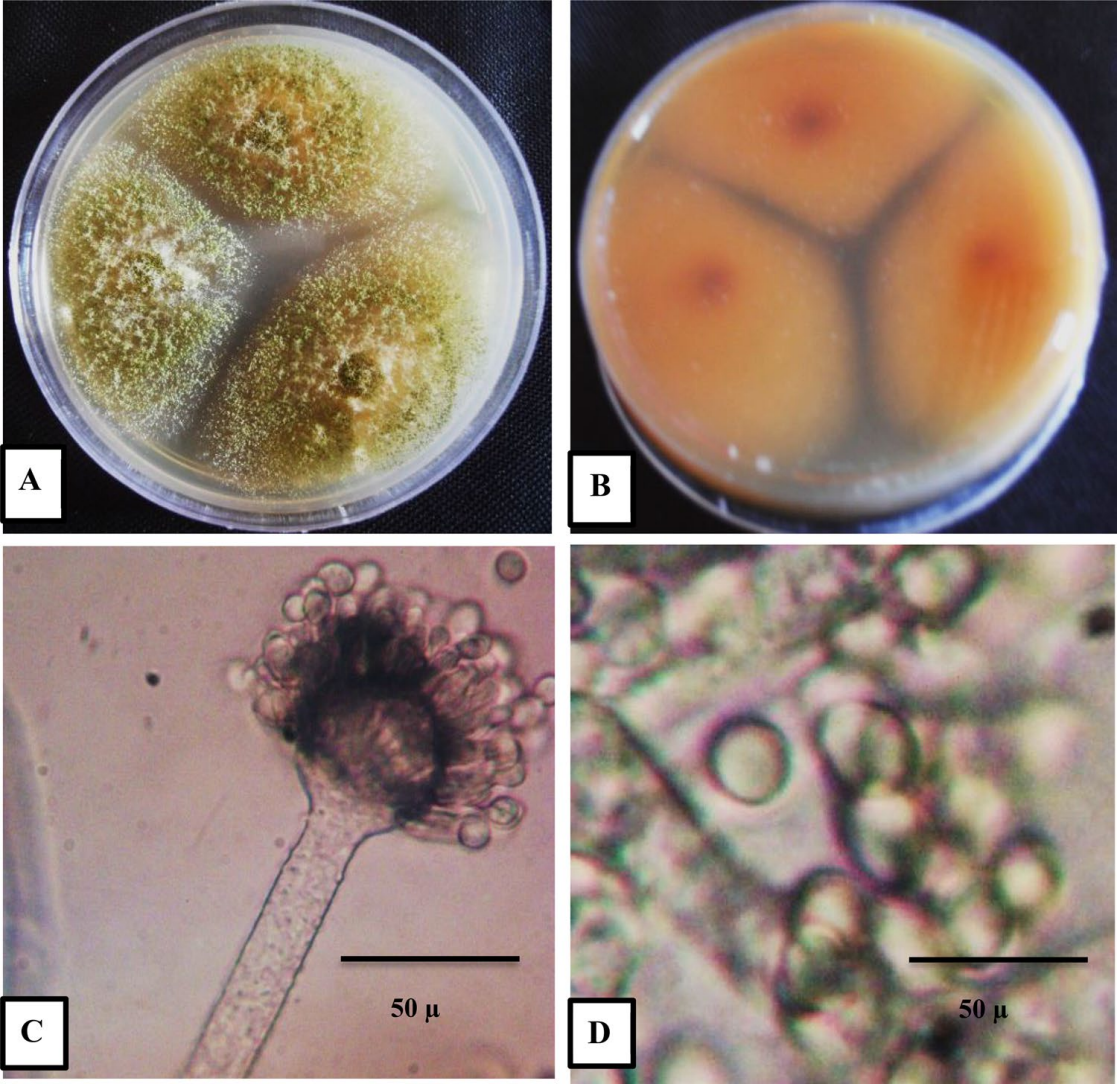
Morphological characters of *Aspergillus flavus*; (**A**) colony on PDA, (**B**) reverse colony on PDA, (**C**) conidiophore and conidial head mag 10 × 40, and (**D**) conidia mag 20 × 40.

Fungal 2 morphologically identified as *Aspergillus flavus*. On PDA colonies After three days, the diameter is 30 to 40 mm, flat, sparse to moderately dense, velutinous at least in the margins, and frequently floccose in the center (Fig. 8A). The reverse colony is shown in Fig. 8B, with the typical orange, dark yellow, and deep orange color in the middle. Pale brown, rough-walled conidiophores borne from subsurface or surface hyphae; globose to sub-globose vesicles fertile over 75% of the surface, usually carrying both metulae and phialides (Fig. 8C). Conidia in Fig. 8D are coarsely roughened, spherical to subspheroidal, and have relatively thin walls.

**Fig. 9:**
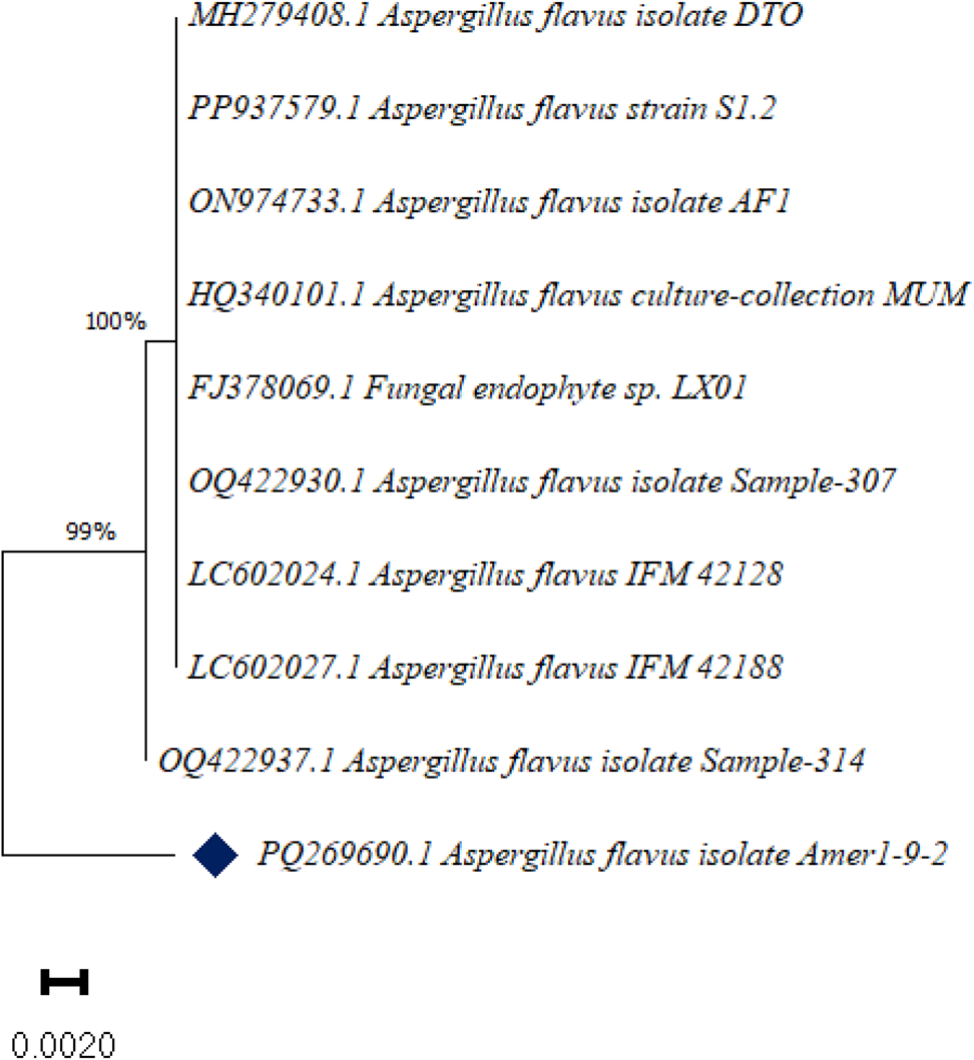
Phylogenetic tree of *Aspergillus flavus*. The evolutionary history was inferred using the Neighbor-Joining method with distances calculated by the Maximum Composite Likelihood method, expressed as base substitutions per site. Branch lengths reflect these distances. Proportions of unambiguous sites are indicated at internal nodes. All evolutionary analyses were performed using MEGA11 software.

To confirm the morphological identification, molecular identification was conducted. Subsequently, the strain was deposited in the NCBI with accession number PQ269690.1. Its similarities to *Aspergillus flavus* isolate DTO 213-I2 (MH279408.1), *Aspergillus flavus* strain S1.2 (PP937579.1), *Aspergillus flavus* isolate AF1 (ON974733.1*)*,* Aspergillus flavus* culture-collection MUM (HQ340101.1), Fungal endophyte sp. LX01 (FJ378069.1), *Aspergillus flavus* isolate Sample-307 (OQ422930.1), *Aspergillus flavus* IFM 42,128 (LC602024.1), *Aspergillus flavus* IFM 42,188 (LC602027.1), *Aspergillus flavus* isolate Sample-314 (OQ422937.1) were 98.23% (Fig. 9; Table 2S).

### Identification of active metabolites by GC mass analysis

**Fig. 10:**
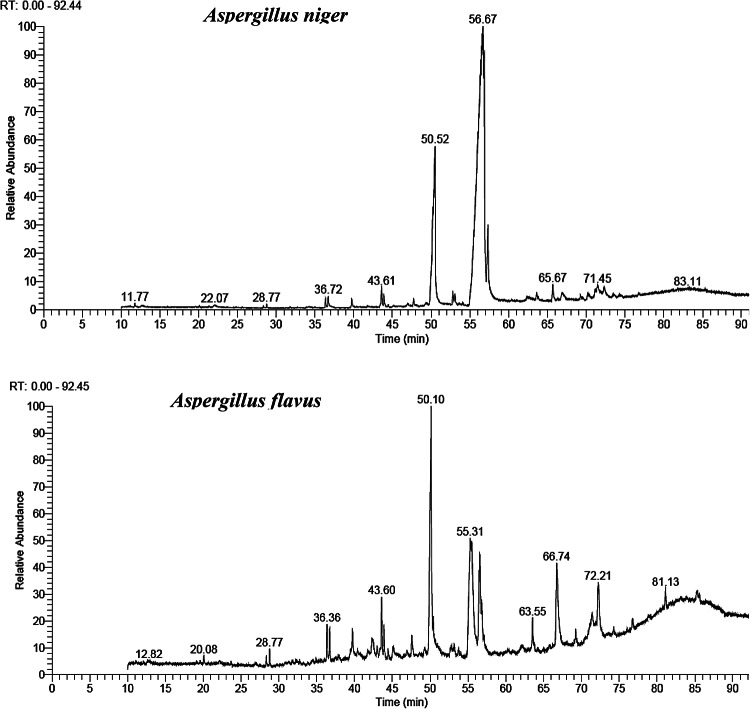
Gas Chromatography-Mass Spectrometry (GC-MS) analysis of bioactive compounds in *Aspergillus niger* and *Aspergillus flavus.*

*Aspergillus niger* yielded 0.3 g per liter of active secondary metabolites, while *Aspergillus flavus* produced 0.5 g per liter, highlighting a higher metabolite output from *A. flavus*. *A. niger* and *A. flavus* were found to contain 12 and 22 compounds, respectively, according to GC-MS analysis. In *A. niger* the top eight compounds, in terms of peak area percentage, were considered major compounds and were as follows: Oleic Acid (26.18%), Hexadecanoic Acid (21.97%), 9,12-Octadecadienoic Acid (Z, Z) (13.81%), Octadecanoic Acid (12.10%), 9,12-Octadecadienoic Acid, Methyl Ester (6.98%), Docosane (4.88%), 3-Hydroxypropyl Palmitate, derivative (3.46%), and Hexadecane (1.64%) (Table 1; Fig. 10).

In *A. flavus* the top ten compounds, in terms of peak area percentage, were considered major compounds and were as follows: Hexadecanoic Acid (22.42%), 9,12-Octadecadienoic Acid (Z, Z) (19.57%), Octadecanoic Acid (10.30%), Hexadecanoic acid, 2-hydroxy-1-(hydroxy methyl)ethyl ester (8.47%), Octadecanoic acid, 2-hydroxy-1- hydroxy methyl ethyl ester (5.03%), 1-Eicosanol (4.19%), Hahnfett (3.50%), Hexadecane (2.72%), 1-Hexadecanol (2.53%), Butyl6,9,12-hexadecatrienoate (2.41%) (Table 1; Fig. 10).

**Table 1:** GC mass analysis of *Aspergillus Niger* and *Aspergillus flavus*.

No	Compounds	RT (min)	Peak area %		Activity	References
			Fungal strains			
			A. niger	A. flavus		
1	Dotriacontane	20.08	-	0.54	Antifungal activity	^26^
2	Nonadecene	28.35	-	0.71	Insecticidal activity	^27^
3	Tetradecane	28.77	-	1.17	Antibacterial and insecticidal activity	^28^
4	1-Hexadecanol	36.36	1.59	2.53	Insecticidal, mosquito larvicides,, antimicrobial, antioxidant, and anticancer activity	^29^
5	Hexadecane	36.72	1.64	2.72	Antibacterial and insecticidal	^28^
6	2,2-dideutero octadecanal	39.72	-	1.77	Insecticidal activity	^30^
7	Dodecyl acrylate	39.73	1.54	-	Surfactant	^31^
8	Butyl6,9,12-hexadecatrienoate	42.36	-	2.41	Antimicrobial and antioxidant activities	^32^
9	Lactaropallidin	43.00	-	0.91	Antibacterial, fungicide, and anti-inflammatory	^33^
10	Docosane	43.19	4.88	-	Insecticidal, antioxidant activity, and antimicrobial	^34^
11	1-Eicosanol	43.60	-	4.19	Insecticidal, antimicrobial, and insect repellent,	^35^
12	9-Octadecenoic acid (z)	44.43	-	0.73	Insecticidal activity	^36^
13	11,14-Eicosadienoic acid, methyl ester	45.09	-	1.41	Insecticidal activity	^37^
14	Erucic acid	47.56	-	1.71	Antimalarial activity, and insecticidal potential	^38^
15	Methyl-9,9,10,10-d- 4-octadecanoate	47.71	-	0.80	Insecticidal activity	^39^
16	Pentadecanoic acid, 14-methyl-,methyl ester	47.73	1.37	-	Insecticidal activity	^40^
17	Hahnfett	49.25	-	3.50	Nematicidal activity	^41^
18	Hexadecanoic acid	50.52	21.97	22.42	Insecticidal activity , hemolytic, pesticide, flavor, and antioxidant	^42^
19	9,12-Octadecadienoic acid, methyl ester	52.79	6.98	-	Insecticidal activities against *Aedes aegypti*, aphids in oilseed rape, cotton insect pests	^42,43^
20	Isochiapin B	53.20	-	1.40	Pesticidal activity, anti-insect and antitumor.	^44^
21	Oleic Acid	55.67	26.18	1.59	Mosquito repellent, mosquito larvicidal activity, and ovicidal activity.	^45^
22	9,12-Octadecadienoic acid (Z, Z)	56.60	13.81	19.57	Anti-cancer and pesticide	^46^
23	Octadecanoic acid	57.33	12.10	10.30	Insecticidal against agriculture and medical insects	^47^
24	2,2-1-Docosanol	63.59	1.36	-	Antiviral activity	^48^
25	3-Hydroxypropyl palmitate, TMS derivative	65.67	3.46	-	insecticidal activity	^49^
26	Hexadecanoic acid, 2-hydroxy-1-(hydroxy methyl)ethyl ester	66.74	-	8.47	Antibacterial activity	^50^
27	17-Pentatriacontene	69.22	-	1.07	Insecticidal, and anti-microbial activity	^51^
28	Octadecanoic acid, 2-hydroxy-1- hydroxy methyl ethyl ester	72.21	-	5.03	Hepatoprotective activities	^52^

## Discussion

The present study aimed to investigate the bioactive potential of endophytic fungi isolated from *Olea europaea* leaves, focusing on their metabolite profiles and larvicidal activities. The identification of fungal endophyte isolates was carried out using a combination of morphological and molecular techniques, ensuring accurate taxonomic classification. The results of this study demonstrate that the most potent insecticidal activity were Extract 1 and Extract 2 isolates. The morphological and molecular identification of these isolates provides a robust foundation for understanding their taxonomic classification and phylogenetic relationships, which are critical for further exploration of their biocontrol potential. Morphologically, *Aspergillus niger*(Extract 1) was characterized by rapid growth on PDA, reaching 30 mm in diameter within three days, with colonies displaying a distinctive black color and pale yellow reverse. The conidiophores, vesicles, and spiny-walled conidia observed in this study are consistent with the typical features of *A. niger* described in the literature. Molecular identification further confirmed the strain’s identity, with a 98.94% similarity to *Aspergillus niger* related species, as evidenced by the phylogenetic analysis. The deposition of the strain in the NCBI database (accession number PQ269689) provides a valuable resource for future comparative studies. Similarly, *Aspergillus flavus*(Extract 2) exhibited morphological features typical of the species, including velutinous colonies with orange to dark yellow reverse coloration, rough-walled conidiophores, and coarsely roughened conidia. These characteristics are consistent with previous descriptions of *A. flavus*^53^. Molecular analysis revealed a 98.23% similarity to *Aspergillus flavus* strains and related taxa, further validating the morphological identification. The deposition of this strain in the NCBI database (accession number PQ269690) enhances its accessibility for future research and underscores its potential as a source of bioactive compounds.

The identified isolates recorded high larval mortality, and larval mortality increased significantly with higher concentrations of the tested metabolites. This finding aligns with previous studies that have shown that low concentrations of fungal metabolites often exhibit sublethal effects insufficient to induce significant mortality in target organisms^54^. As the concentration increased to the range of 43.75–100.0 µg/mL, a slight rise in mortality was observed, though the median remained low, suggesting only marginal efficacy. This trend is consistent with the gradual onset of toxicity often observed in bioassays involving fungal-derived compounds. The observed variability in mortality at higher concentrations may be attributed to factors such as larval age, physiological condition, or genetic variability, which can influence susceptibility to toxic agents^55^. The dose-response curves provide further insights into the larvicidal potency of the tested extracts. These findings are consistent with previous research demonstrating the efficacy of fungal metabolites in disrupting insect physiology and development. The tighter confidence intervals for *A. niger*, and *A flavus* suggest more consistent toxic effects, likely due to the presence of potent bioactive compounds. In contrast, Extract 3 and Extract 4 displayed weaker toxic effects, with higher LC₅₀ values (228.09 and 232.91 µg/mL, respectively) and more gradual dose-response slopes. The wider confidence intervals for these extracts indicate greater variability in their impact, which may reflect differences in metabolite composition or larval susceptibility. The differential larvicidal potency observed among the extracts highlights the importance of characterizing the chemical constituents responsible for their bioactivity. Previous studies have shown that endophytic fungi produce a diverse array of secondary metabolites, including alkaloids, terpenoids, and polyketides, which can exhibit insecticidal properties^9^.

The histological examination of digestive system tissues treated with fungal extracts revealed a clear dose-dependent effect on tissue, cellular integrity, and inflammatory response. These alterations are indicative of severe cellular stress and damage, likely resulting from the cytotoxic effects of the fungal metabolites. The observed nuclear condensation and apoptotic features are consistent with the activation of programmed cell death pathways, which are often triggered by exposure to high concentrations of insecticidal compounds^55^. The extensive structural disruption and loss of cellular cohesion observed at 200 µg/mL further highlight the potent cytotoxic effects of the fungal extracts at elevated doses. The histological changes observed in this study provide valuable insights into the mechanisms underlying the larvicidal activity of the fungal extracts. The dose-dependent disruption of cellular integrity and induction of apoptosis suggest that the metabolites exert their toxic effects by targeting cellular structures and processes essential for larval survival. The observed inflammatory response further highlights the complex interplay between direct cytotoxicity and immune-mediated tissue damage in the overall toxicity of the extracts. The GC mass analysis indicated the presence of active metabolites in the *A. niger* and *A. flavus* extract were linked to a variety of behaviors: insecticidal activity, mosquito repellent, mosquito larvicidal activity, ovicidal, hemolytic, flavor, antioxidant, Hepatoprotective, anti-cancer, insecticidal against agricultural and medical insects, antibacterial and insecticidal activity against *Aedes aegypti*, aphids in oilseed rape, and cotton insect pests. Numerous investigations have demonstrated the potential of oleic acid, Octadecadienoic Acid and Hexadecanoic acid as a low-toxicity natural insecticidal agent for both beneficial organisms and humans^56^. It can incorporate into lipid bilayers, disrupting membrane integrity. It may interfere with the respiratory processes of insects, potentially disrupting energy metabolism and deterring insect feeding and oviposition^57^. Eicosanol, can interfere with growth-control hormones, causing aberrant growth and development, as well as hindering processes like molting and reproduction. Eicosanol may be more effective as an insecticide by lowering bacteria levels and causing damage to the insect or altering its normal gut flora due to its antimicrobial activity against various microorganisms. Lipid membranes are susceptible to Eicosanol incorporation, which could compromise their integrity and result in cell leakage and death^58^.

The findings of this study are significant in the context of developing sustainable and eco-friendly alternatives to chemical insecticides for mosquito control. The integration of endophytic fungi into pest management strategies offers a promising approach, as these microorganisms produce a diverse array of bioactive compounds with potential insecticidal properties. The presence of hydrocarbons such as docosane and hexadecane suggests a role in volatile organic compound (VOC) signaling, which may contribute to interspecies interactions and ecological adaptation. Endophytic fungi such as *A. niger* and *A. flavus* are known for producing bioactive secondary metabolites with potential applications in controlling pathogens. When carefully processed and applied, their extracts can offer environmentally friendly alternatives to synthetic chemicals, minimizing pollution and preserving biodiversity^59^. Studies have shown that certain metabolites from these fungi, including organic acids exhibit biological activity without persistent toxicity in the environment^60^.

## Conclusion

Four endophytic fungal isolates were carried out from *Olea europaea* and tested against mosquitoes. Toxicity assays demonstrated a strong insecticidal effect, with Extract 1 and Extract 2 exhibiting the highest larvicidal activity (LC₅₀ values of 148.36 and 153.36 µg/mL, respectively), whereas Extracts 3 and 4 showed significantly weaker effects. Histopathological analysis further revealed that higher extract concentrations (150–200 µg/mL) caused severe tissue disorganization, apoptosis, and inflammatory responses, while lower concentrations (25–50 µg/mL) induced minimal cytotoxicity. The most potent fungal isolates were identified morphology as *Aspergillus niger*(Extract 1) and *Aspergillus flavus*(Extract 2) and confirmed through molecular characterization, showing high similarity to known strains in the NCBI database. GC-MS analysis identified major bioactive metabolites, including oleic acid, hexadecanoic acid, and 9,12-octadecadienoic acid, which are associated with insecticidal activities. These compounds likely disrupt insect membrane integrity and energy metabolism, contributing to their larvicidal potential. Thus we can suggest the application of *Aspergillus niger* and *Aspergillus flavus* as bio insecticidal agents alternate to harmful chemical insecticidal. Future research should focus on formulating, and optimizing Aspergillus-based bioinsecticides, assessing environmental safety, enhancing metabolite yield, and expanding efficacy studies across mosquito species and developmental stages.

## Electronic supplementary material

Below is the link to the electronic supplementary material.

Supplementary Material 1

## Data Availability

All data supporting the findings of this study are transparently presented within the manuscript. No additional datasets were generated or analyzed beyond those included in the figures and tables, ensuring complete accessibility for review and reproducibility.
