# Supplementary material for: Bioinsecticidal activity of aspergillus-derived endophytes from Olea europaea against Culex pipiens: toxicity, histology, and GC-MS profiling
Source: Sci Rep. 2025 Jul 4;15:23952. doi: 10.1038/s41598-025-07941-3 (PMC12227682; doi:10.1038/s41598-025-07941-3)
Supplement: Supplementary file 1 — Supplementary Material 1 [file 41598_2025_7941_MOESM1_ESM.doc]

Table 1 S fungi related to *Aspergillus niger* isolate Amer1-9-1 PQ269689.1

| Fungi | Accession IDs |
| --- | --- |
| *Aspergillus niger* strain KAML02 | KC119204.1 |
| *Aspergillus niger* isolate MSFW | OR668932.1 |
| *Aspergillus niger* isolate MSSFW | OR668928.1 |
| *Aspergillus niger* 18S | KF304798.1 |
| *Aspergillus niger* 28S rRNA | XR009468006.1 |
| *Aspergillus welwitschiae* strain CBS | OL711714.1 |
| *Aspergillus niger* strain NJA-1 | KJ365316.1 |
| *Aspergillus niger* strain KAML02 | KC119204.1 |
| *Aspergillus niger* contig An03c0100 | AM270051.1 |
| *Aspergillus niger* contig An03c0110 | AM270052.1 |

Table 2 S fungi related to *Aspergillus flavus strain Amer1-9-2* PQ269690.1

| Fungi | Accession IDs |
| --- | --- |
| *Aspergillus flavus* strain S1.2 | PP937579.1 |
| *Aspergillus flavus* isolate AF1 | ON974733.1 |
| *Aspergillus flavus* isolate DTO | MH279408.1 |
| *Aspergillus flavus* culture collection MUM | HQ340101.1 |
| Fungal endophyte sp*.* LX01 | FJ378069.1 |
| *Aspergillus flavus* isolate Sample-307 | OQ422930.1 |
| *Aspergillus flavus* isolate sample-314 | OQ42293.1 |
| *Aspergillus flavus* IFM 42188 | LC602027.1 |
| *Aspergillus flavus* IFM 42128 | LC602024.1 |
